# Transperitoneal vs retroperitoneal minimally invasive partial nephrectomy: comparison of perioperative outcomes and functional follow-up in a large multi-institutional cohort (The RECORD 2 Project)

**DOI:** 10.1007/s00464-020-07919-4

**Published:** 2020-08-27

**Authors:** Francesco Porpiglia, Andrea Mari, Daniele Amparore, Cristian Fiori, Alessandro Antonelli, Walter Artibani, Pierluigi Bove, Eugenio Brunocilla, Umberto Capitanio, Luigi Da Pozzo, Fabrizio Di Maida, Paolo Gontero, Nicola Longo, Giancarlo Marra, Bernardo Rocco, Riccardo Schiavina, Claudio Simeone, Salvatore Siracusano, Riccardo Tellini, Carlo Terrone, Donata Villari, Vincenzo Ficarra, Marco Carini, Andrea Minervini, The RECORD 2 Project, Vincenzo Altieri, Francesco Berardinelli, Antonio Celia, Elisabetta Costantini, Alberto Diminutto, Mario Falsaperla, Matteo Ferro, Maria Furlan, Gaetano Grosso, Alessandro Larcher, Vincenzo Li Marzi, Francesco Montorsi, Andrea Polara, Angelo Porreca, Riccardo Rizzetto, Marco Roscigno, Luigi Schips, Cesare Selli, Sergio Serni, Alchiede Simonato, Carlo Trombetta, Giuseppe Vespasiani, Alessandro Volpe

**Affiliations:** 1 Division of Urology, Department of Oncology- School of Medicine, University of Turin, San Luigi Hospital, Orbassano, Turin, Italy; 2 Department of Urology, Unit of Oncologic Minimally-Invasive Urology and Andrology, Careggi Hospital, University of Florence, Florence, Italy; 3 Department of Urology, Spedali Civili Hospital, University of Brescia, Brescia, Italy; 4 Department of Urology, Azienda Ospedaliera Universitaria Integrata (A.O.U.I.), Verona, Italy; 5 Department of Urology, University Hospital of Tor Vergata, Rome, Italy; 6 Department of Urology, University of Bologna, Bologna, Italy; 7 Department of Experimental, Diagnostic, and Specialty Medicine, University of Bologna, Bologna, Italy; 8 Unit of Urology, Division of Experimental Oncology, URI-Urological Research Institute, Vita-Salute San Raffaele University, IRCCS San Raffaele Scientific Institute, Milan, Italy; 9 Department of Urology, Papa Giovanni XXIII Hospital, Bergamo, Italy; 10 Division of Urology, Department of Surgical Sciences, San Giovanni Battista Hospital, University of Studies of Torino, Turin, Italy; 11 Department of Urology, University Federico II of Naples, Naples, Italy; 12 Department of Urology, Fondazione IRCCS Ca’ Granda‚ Ospedale Maggiore Policlinico‚ Policlinico‚ University of Milan, Milan, Italy; 13 Department of Urology, University of Modena and Reggio Emilia, Modena, Italy; 14 Department of Urology, University of Genova, Genova, Italy; 15 Department of Urology, Unit of Urological Minimally Invasive Robotic Surgery and Renal Transplantation, Careggi Hospital, University of Florence, Florence, Italy; 16 Department of Human and Paediatric Pathology, Gaetano Barresi, Urologic Section, University of Messina, Messina, Italy; 17 Department of Urology, Careggi Hospital, San Luca Nuovo, University of Florence, Florence, Italy

**Keywords:** Renal cell carcinoma, Minimally invasive partial nephrectomy, Transperitoneal, Retroperitoneal, Surgical approach

## Abstract

**Background:**

Aim of this study was to evaluate and compare perioperative outcomes of transperitoneal (TP) and retroperitoneal (TR) approaches in a multi-institutional cohort of minimally invasive partial nephrectomy (MI-PN).

**Material and methods:**

All consecutive patients undergone MI-PN for clinical T1 renal tumors at 26 Italian centers (RECORd2 project) between 01/2013 and 12/2016 were evaluated, collecting the pre-, intra-, and postoperative data. The patients were then stratified according to the surgical approach, TP or RP. A 1:1 propensity score (PS) matching was performed to obtain homogeneous cohorts, considering the age, gender, baseline eGFR, surgical indication, clinical diameter, and PADUA score.

**Results:**

1669 patients treated with MI-PN were included in the study, 1256 and 413 undergoing TP and RP, respectively. After 1:1 PS matching according to the surgical access, 413 patients were selected from TP group to be compared with the 413 RP patients. Concerning intraoperative variables, no differences were found between the two groups in terms of surgical approach (lap/robot), extirpative technique (enucleation vs standard PN), hilar clamping, and ischemia time. Conversely, the TP group recorded a shorter median operative time in comparison with the RP group (115 vs 150 min), with a higher occurrence of intraoperative overall, 21 (5.0%) vs 9 (2.1%); *p* = 0.03, and surgical complications, 18 (4.3%) vs 7 (1.7%); *p* = 0.04. Concerning postoperative variables, the two groups resulted comparable in terms of complications, positive surgical margins and renal function, even if the RP group recorded a shorter median drainage duration and hospital length of stay (3 vs 2 for both variables), *p* < 0.0001.

**Conclusions:**

The results of this study suggest that both TP and RP are feasible approaches when performing MI-PN, irrespectively from tumor location or surgical complexity. Notwithstanding longer operative times, RP seems to have a slighter intraoperative complication rate with earlier postoperative recovery when compared with TP.

**Electronic supplementary material:**

The online version of this article (10.1007/s00464-020-07919-4) contains supplementary material, which is available to authorized users.

The incidental detection of renal masses increased over the last decades due to the expanding use of imaging diagnosis performed for other reasons [1]. Alongside with this occurrence, kidney surgery has undergone both theoretical and technical developments [2, 3]. Firstly, the indication for partial nephrectomy (PN) in elective cases has been expanded widely, being well demonstrated the advantages of partial over radical surgery in T1 tumors in terms of functional preservation with similar oncological safety [4–6]. On the other hand, the technological improvements of the last 30 years paved the way for the gradually increased employment of minimally invasive approaches, first with the advent of laparoscopy and then with robotics, progressively minimizing the impact and invasiveness of surgical procedures [7–9].

The surgical access, either trans or retroperitoneal (TP or RP), plays a key role in kidney surgery, since it meaningfully influences the comfort of the procedure as well as perioperative outcomes [10, 11]. To date, the choice between TP or RP access has been traditionally influenced by tumor- (location and surgical complexity) and patient-related features (previous abdominal surgery, presence of abundant abdominal fat) [12, 13]. However, in the last few years, the ongoing improvements of computer-enhanced technology have provided the necessary means for surgeons to assimilate minimally invasive surgical options into their everyday surgical practice and overcome the surgical limitation related to conventional surgery. In this light, robotic surgery made easier to perform even complex surgical procedures with both accesses thanks to an improved ergonomic position for the surgeon, three-dimensional vision of the operating field and augmented maneuverability of the instruments due the EndoWrist® technology.[14].

Nevertheless, to date, the final choice between TP or RP approach still remains strictly influenced by the surgeon preference and the advantages of different surgical access during laparoscopic or robotic PN has not been clearly highlighted yet [15, 16].

To fill this gap, the aim of the current study was to evaluate and compare the perioperative outcomes in patients treated with TP and RP minimally invasive PN for clinical T1 renal tumors in a large multi-institutional study (The RECORD 2 project).

## Materials and methods

The Italian REgistry of COnservative and Radical Surgery for cortical renal tumor Disease (RECORD 2 Project) is a prospective observational multicenter project promoted by the Italian Society of Urology (SIU). This study was approved by the local ethics committee, and written informed consent was collected for all the patients. Overall 4325 consecutive patients undergone renal surgery for cortical renal tumors at 26 urological Italian centers between January 1, 2013, and December 31, 2016, were included. For each patient, anthropometric and preoperative data, imaging, indications and co-morbidities, intraoperative data, postoperative data, histological analysis, follow-up were collected in an e-form central database to limit missing or wrong data inputs. Comorbidity status was evaluated by Charlson comorbidity index (CCI), physical status (PS) by the American Society of Anesthesiologists (ASA) classification system. Surgical indications were defined as elective (unilateral lesion with healthy contralateral kidney), relative (presence of diabetes, hypertension or lithiasis that could potentially affect kidney function in the future) and absolute (bilateral tumors, multiple tumors, moderate to severe CKD or tumors involving an anatomically or functionally solitary kidney). Center experience was evaluated as number of PN/year and PN/RN ratio per year. Surgical approach and type of resection were chosen according to surgeons’ preference and centers availability.

For the purpose of this study, only patients undergone minimally invasive PN (mi-PN) for clinical T1 renal tumors were included at a first stage. Therefore, 1712 patients treated with radical nephrectomy and 886 patients treated with open PN, 47 patients with cT ≥ 2 and 11 patients with missing data undergone minimally invasive PN were excluded and only patients undergone minimally invasive TP (*n* = 1256) and RP (*n* = 413) from 21 centers were included.

### Statistical analysis

A propensity score matching was performed on the selected cohort to settle on two cohorts of patients undergone TP and RP MI-PN with comparable preoperative clinical features [17]. The matching was carried out with a 1:1 ratio with respect to the surgical access (413 TP vs 413 RP mi-PN) with a C statistic of 0.67 adjusting for the variables: age, gender, baseline eGFR, surgical indication, clinical diameter, ECOG and ASA PS scores, tumor location and PADUA score. The difference between the two groups is plotted in Supplementary Fig. 1. The Student t-test and the Mann–Whitney-*U* test were used to compare continuous to categorical variables and the Pearson’s Chi-square test was used to compare two categorical variables. Statistical significance was set at *p* < 0.05. All reported *p* values were two-sided. Analyses were carried out with RStudio graphical interface v.0.98 for R software environment v.3.0.2, using the packages MatchIt, rms and histbackback, and with STATA v.14.1 (StataCorp LP, College Station, TX).

**Fig. 1:**
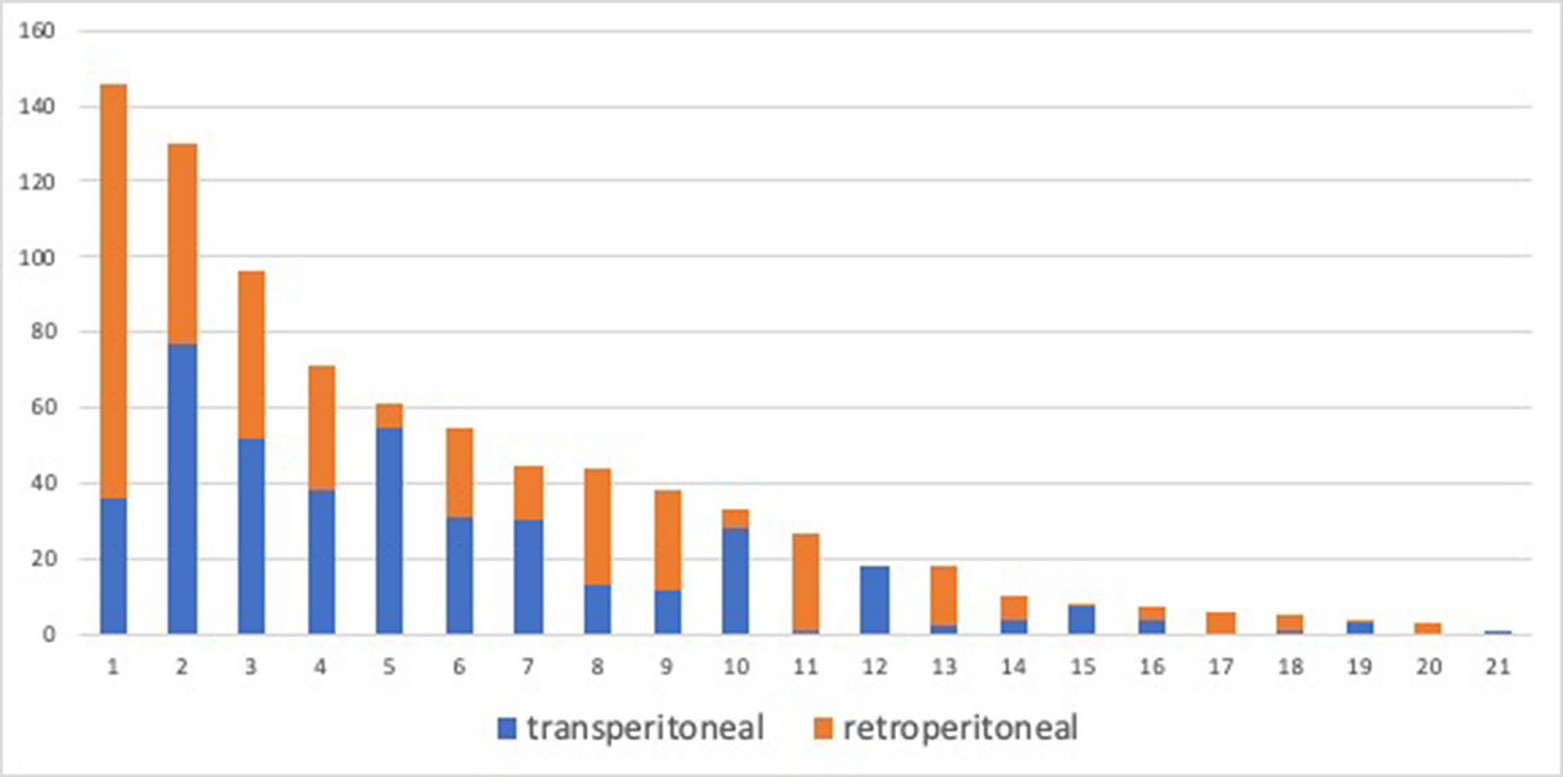
Distribution of transperitoneal and retroperitoneal surgical procedures according to each single centre after propensity score matching analysis

## Results

### Patients and tumors features

Preoperative tumor and patient features of the selected cohorts after propensity score matching are reported in Table 1. After propensity score matching, all the variables were comparable between the two groups. In detail, patients included in the TP and RP cohorts were males in 65.6% and 61.8% of the cases (*p* = 0.67), respectively. Patients had a median (interquartile range [IQR]) age of 64.8 (56.2–72.4) and 64.6 (55.9–72.3) years (*p* = 0.71), and a median (IQR) BMI of 25.9 (23.9–28.7) and 25.8 (23.7–28.4) in the TP and RP group (*p* = 0.95), respectively. The CCI score and the ASA PS score were 1 (0–2) and 2 (2–3) for both groups. Patients had relative and absolute indication for surgery in 20.8% and 3.7% of TP group versus 24.9% and 5.5% of the RP group (*p* = 0.24).

**Table 1:** Comparison of the preoperative characteristics of 413 patients treated with retroperitoneal minimally invasive partial nephrectomy with selected matched 413 patients treated with transperitoneal minimally invasive partial nephrectomy for renal tumors within the RECORD 2 project

RECORD 2 project—preoperative tumor and patient data	Surgical access				*p* value
	Transpertioneal (*n* = 413)		Retroperitoneal (*n* = 413)		
Gender, *n*. %					
Male	271	65.6%	280	67.8%	0.67
Female	142	34.4%	133	32.2%	
Age (years), median IQR	64.8	56.2–72.4	64.6	55.9–72.3	0.71
BMI, median IQR	25.9	23.9–28.7	25.8	23.7–28.4	0.95
ECOG score, *n*. %					
0	311	75.3%	315	76.3%	0.69
≥ 1	92	24.6%	98	23.7%	
CCI score, median IQR	1	0–2	1	0–2	0.71
ASA PS score, median IQR	2	2–3	2	2–3	0.82
Indication, *n*. %					
Elective	312	75.5%	287	69.6%	0.24
Relative	86	20.8%	103	24.9%	
Absolute	19	3.7%	23	5.5%	
Clinical T, *n*. %					
T1a	330	79.9%	331	80.1%	0.82
T1b	83	20.1%	82	19.9%	
Tumor side, *n*. %					
Right	202	48.9%	194	47.0%	0.93
Left	211	51.1%	219	53.0%	
Clinical diameter, median IQR	3.2	1.9–4.6	3.1	1.8–4.5	0.63
Tumor site, *n*. %					
Mesorenal	271	65.6%	278	67.3%	0.18
Polar	142	34.4%	135	32.7%	
Tumor growth pattern, *n*. %					
≥ 50% Exophytic	229	55.4%	240	58.1%	0.41
< 50% Exophytic	160	38.7%	147	35.6%	
Entirely endophytic	24	5.8%	26	6.3%	
PADUA Score, median IQR	7.0	7.0–8.0	7.0	7.0–8.0	0.69
PADUA complexity group, n. %					
Low (6–7)	244	59.1%	251	60.8%	0.78
Medium (8–9)	155	37.5%	146	35.4%	
High (≥ 10)	14	3.4%	16	3.9%	
Tumor localization, *n*. %					
Lateral margin	139	33.7%	143	34.6%	0.26
Medial margin	49	11.8%	46	11.3%	
Anterior face	41	9.9%	37	9.0%	
Posterior face	183	44.3%	187	45.0%	
Peri-hilar	1	0.2%	0	0%	
Preoperative hemoglobin, mean SD	14.5	13.4–15.3	14.2	13.3–15.2	0.83
Preoperative creatinine (mg/dl), mean SD	0.9	0.80–1.00	0.9	0.80–1.10	0.19
Preoperative eGFR, median IQR	85.1	70.3–96.6	82.8	66.8–99.5	0.67

A clinical T1a (cT1a) and cT1b stage were reported in 79.9% and 20.1% of TP while in 80.1% and 19.9% of the RP patients (*p* = 0.82), respectively. The median (IQR) PADUA score was 7 (7–8) for both groups (*p* = 0.69), with PADUA ≥ 10 registered in 3.4% of TP and 3.9% of RP patients, respectively. The propensity score matching evened the differences also in terms of tumor location: the percentage of tumors located on the lateral margin, medial margin, anterior face and posterior face were 33.7% vs 34.6%, 11.8% vs 11.3%, 9.9% vs 9.0% and 44.3% vs 45.0% for TP and RP groups, not considering the surgical access. At baseline the median (IQR) values of preoperative creatinine were 0.9 mg/dl (0.8–1) and 0.9 mg/dl (0.8–1.1), with an eGFR of 85.1 ml/min/m^2^ (70.3–96.6) and 82.8 ml/min/m^2^ (66.8–99.5) for TP and RP cohort, respectively.

### Surgical features

The surgical features of the two cohorts after propensity score matching are summarized in Table 2.

**Table 2:** Comparison of the centers data and the intraoperative characteristics of 413 patients treated with retroperitoneal minimally invasive partial nephrectomy with selected matched 413 patients treated with transperitoneal minimally invasive partial nephrectomy for renal tumors within the RECORD 2 project

RECORD 2 project—intraoperative center and surgical data	Surgical access		*p* value
	Transpertioneal (*n* = 413)	Retroperitoneal (*n* = 413)	
PN/RN ratio, median (IQR)	66.6% (50.6–78%)	74.0% (66.6–87.9%)	< 0.0001
Number of PN/year of the center of treatment, median (IQR)	56 (35–79)	71 (44–84)	< 0.0001
Center volume, *n* (%)			
> 30 PN/year	313 (75.8%)	331 (80.1%)	0.001
> 50 PN/year	256 (62.0%)	315 (76.3%)	0.001
Surgical approach			
Laparoscopic	281 (68.0%)	279 (67.5%)	0.67
Robotic	132 (32.0%)	134 (33.5%)	
Technique, *n* (%)			
Enucleation	173 (41.9%)	169 (40.9%)	0.77
Standard PN	240 (58.1%)	244 (59.1%)	
Hilar clamping, *n* (%)			
Not performed	198 (47.9%)	213 (51.6%)	0.29
Performed	215 (52.1%)	200 (48.4%)	
Ischemia time(min), median (IQR)	19 (14–24)	20 (15–25)	0.14
EBL (cc), median (IQR)	100 (100–200)	150 (100–143)	0.10
Extended peritoneum opening, *n* (%)	32 (7.7%)	–	–
Intraoperative time, median (IQR)	115 (96–130)	150 (120–180)	< 0.0001
Intraoperative overall complications, *n* (%)	21 (5.0%)	9 (2.1%)	0.03
Intraoperative surgical complications, *n* (%)	18 (4.3%)	7 (1.7%)	0.04
Vascular lesion, *n* (%)	9 (2.1%)	4 (0.8%)	–
Spleen injury, *n* (%)	2 (0.4%)	–	–
Conversion to open procedure, *n* (%)	4 (0,8%)	2 (0.4%)	–
Bleeding from renal resection bed, *n* (%)	3 (0.2%)	1 (0.2%)	–
Intraoperative medical complications, *n* (%)	3 (0.7%)	2 (0.5%)	0.64
Miocardial infarction, *n* (%)	2 (0.4%)	1 (0.2%)	–
Arrytmias, *n* (%)	1 (0.2%)	1 (0.2%)	–

Overall, 12/21 (57.1%) and 6/21 (28.6%) centers performed > 30 and > 50 mi-PNs per year, respectively. An overview of the distribution of surgical accesses for each center is shown in Fig. 1. Patients undergone RP were treated in centers performing a median (IQR) of 71 (44–84) PNs/year, while the matched patients undergone TP underwent surgery in centers with a median of 56 (35–79) PN/year (*p* < 0.0001). Specifically, in the RP cohort, 331 (80.2%) and 315 (76.3%) patients were treated in centers performing > 30 and > 50 PNs/year, while of the TP cohort only 313 (75.8%) and 256 (62.0%) patients underwent surgery in centers performing > 30 and > 50 PNs/year (*p* < 0.001). Moreover, the median PN/RN ratio was 74.0% (66.6–87.9%) and 66.6% (50.6–78%) for RP and TP groups, respectively (*p* < 0.001).

Laparoscopic PN (LPN) and robot-assisted PN (RAPN) were planned in 281 (68.0%) and 132 (32.0%) cases with TP access versus 279 (67.5%) and 134 (33.5%) cases with RP access, respectively (*p* = 0.67).

Considering the hilar clamping approach, in 47.9% of the TP and 51.6% of the RP surgeries, renal pedicle was not clamped. When hilar clamping was performed, the median (IQR) ischemia time was 19 (14–24) and 20 (15–25) min for the two groups (*p* = 0.14). Simple enucleation was performed in 41.9% of the TP and 40.9% of the RP, respectively (*p* = 0.77).

Median EBL for the two groups were similar (100 ml and 150 ml, *p* = 0.10). Conversely, the RP group had shorter median intraoperative (skin to skin) time compared the TP group (115 vs 150 min, *p* < 0.0001).

The intraoperative overall complications rate (5% vs 2.1%, *p* = 0.03) and the intraoperative surgical complications rate (4.3% vs 1.7%, *p* = 0.04) were higher in the TP compared to the RP group. Conversely, no differences between the groups were recorded in terms of intraoperative medical complications (0.7% vs 0.5%, *p* = 0.64).

### Postoperative and functional features

Postoperative and functional outcomes of the two cohorts after propensity score matching are shown in Table 3. No differences were found in terms of postoperative complication rates between the two groups, considering both surgical and medical ones (7% vs 6.1% and 3.9% vs 3.4% in TP and RP group, *p* = 0.57 and *p* = 0.48, respectively). Stratifying the surgical complications according to Clavien-Dindo classification [20], 4.1% and 3.4% Clavien > 2 complications were recorded in TP and RP groups, respectively (*p* = 0.57).

**Table 3:** Comparison of the postoperative outcomes of 413 patients treated with retroperitoneal minimally invasive partial nephrectomy with selected matched 413 patients treated with transperitoneal minimally invasive partial nephrectomy for renal tumors within the RECORD 2 project

RECORD 2 project—report of early postoperative and functional follow-up outcomes	Surgical access		*p* value
	Transpertioneal (n = 413)	Retroperitoneal (n = 413)	
Surgical postoperative complications, *n* (%)	29 (7%)	25 (6.1%)	0.57
Surgical clavien 2 postop complications, *n* (%)	12 (2.9%)	11 (2.7%)	
Surgical clavien 3a, postop complications, *n* (%)	11 (2.7%)	10 (2.4%)	
Surgical clavien 3b, postop complications, *n* (%)	3 (0.7%)	4 (1%)	
Surgical clavien 4a, postop complications, *n* (%)	3 (0.7%)	0	
Transfusions, *n* (%)	21 (5.1%)	15 (3.6%)	
Deep vein thrombosis, *n* (%)	1 (0.2%)	0	
Superselective embolization, *n* (%)	8 (2.0%)	7 (1.7%)	
Urinary fistula treated without stenting, *n* (%)	4 (0.8%)	4 (0.8%)	
Urinary fistula treated with stenting or nephrostomy tube, *n* (%)	2 (0.4%)	1 (0.2%)	
Medical postoperative complications, *n* (%)	16 (3.9%)	14 (3.4%)	0.48
Respiratory complications, *n* (%)	8 (2.0%)	8 (2.0%)	
Cardiologic complications, *n* (%)	7 (1.7%)	5 (1.2%)	
Acute pulmonary embolism, *n* (%)	1 (0.2%)	1 (0.2%)	
Drainage duration (days), median (IQR)	3 (2–4)	2 (1–2)	< 0.0001
Time to bowel canalization, median (IQR)	3 (2–5)	2 (1–3)	< 0.0001
Hospital length of stay (days), median (IQR)	3 (3–4)	2 (2–3)	< 0.0001
Malignant tumors, *n* (%)	308 (74.6%)	310 (75.1%)	0.82
Positive surgical margins, *n* (%)	21 (5.1%)	23 (5.6%)	0.73
Preoperative—1st postoperative day Δ hemoglobin (mg/dL), median (IQR)	1.7 (1.1–3.0)	1.5 (0.9–2.7)	0.48
Preoperative—3rd postoperative day Δ hemoglobin (mg/dL), median (IQR)	2.2 (1.4–3.1)	1.8 (1.0–2.9)	0.25
1st POD creatinine (mg/dl), median (IQR)	1.0 (0.8–1.2)	1.0 (0.8–1.3)	0.18
1st POD eGFR, median (IQR)	76 (61.0–89.0)	73 (57.2–89.0)	0.08
Preoperative—1st POD Δ eGFR, median (IQR)	10.3 (0.0–20.8)	10.4 (0.0–22.5)	0.69
Preoperative—3rd POD Δ eGFR, median (IQR)	10.1 (0.0–20.1)	9.0 (0.0–20.7)	0.53
Preoperative—6th month Δ eGFR, median (IQR)	8.7 (0.3–15.9)	8.8 (0.4–15.0)	0.65
Preoperative—12th month Δ eGFR, median (IQR)	9.2 (4.1–21.5)	9.3 (3.4–20.8)	0.67
Preoperative—24th month Δ eGFR, median (IQR)	9.6 (4.4–20.5)	9.8 (4.5–20.0)	0.68

The median (IQR) days of drain was longer in the TP compared to the RP group (3 (2–4) vs 2 (1–2), *p* < 0.0001) as well as time to canalization (3 (2–5) vs 2 (1–3), *p* < 0.0001) and hospital length of stay [3 (3–4) vs 2 (2–3) days, *p* < 0.0001].

Conversely, no differences were recorded between the groups considering the percentage of malignant tumors and the positive surgical margins rate (74.6% vs 75.1% and 5.1% vs 5.6% for TP and RP, respectively).

Regarding the functional outcomes, the two groups resulted comparable in terms of early postoperative median serum creatinine (1 mg/dl, *p* = 0.18) and eGFR levels (76 ml/min/m^2^ and 76 ml/min/m^2^ for TP and RP, *p* = 0.08). Moreover, when considering the median variation of eGFR between the baseline value and each different evaluation considered during the follow-up (3rd POD, 6th month, 12th month, 24th month), no significant differences between TP and RP mi-PN were recorded.

## Discussion

Open PN has traditionally represented the gold standard treatment for the surgical management of localized renal masses amenable to conservative surgery [1, 5]. However, in the last decades minimally invasive approaches in PN have been confirmed as safe, feasible, and effective alternatives to open surgery with several proven advantages such as shorter postoperative pain and, thus, hospital stay and lower complication rate [18–20].

The progressively greater ability of surgeons with minimally invasive approaches led to a significantly rising adoption of RP access [10–12, 15]. The RP access can be extremely challenging from a surgical standpoint due to (1) the limited working space which makes the development of RP space a meticulous and crucial step and (2) the possibility of breaching the peritoneum (3) a slightly harder identification and isolation of the anatomical landmarks. However, the RP access in mi-PN allows to gain a prompt and direct access to renal hilum to reduce operative time and the risk of renal pedicle injury during its isolation [15]. Furthermore, the constraint of the procedure in the retroperitoneum allows to minimize the postoperative ileus and even more significantly reduce the postoperative distress and facilitate an early discharge.

In this study, a large, nation-based and prospectively compiled contemporary dataset was analyzed to compare the perioperative and medium-term functional outcomes in patients with cT1 renal tumors and comparable characteristics at baseline, through a propensity score matching, and undergone TP versus RP mi-PN [21]. We found that RP mi-PN was more significantly performed in centers with higher caseload and a higher attitude to perform conservative surgery. Patients undergone RP mi-PN had a significantly lower rate of overall and surgical intraoperative complications, a lower time of drain maintenance and postoperative hospital stay compared to those patients treated with TP mi-PN. Conversely, intraoperative time was significantly lower in TP mi-PN procedures compared to their counterparts. Furthermore, our results showed comparable outcomes between RP and TP mi-PN in terms of pedicle clamping and ischemia time, postoperative complications, surgical margins, and short- and medium-term renal function recovery.

Our study suggests that both accesses, either TP or RP, are generally feasible and allow comparable perioperative outcomes. In the last decade, many authors faced up to the question whether TP or RP approach is better in performing PN, especially with the diffusion of laparoscopic and robotic approaches. However, no definitive conclusions have been drawn and this seems to still remain a never-ending issue [10–13, 15].

One possible explanation could be that surgical approach is still mainly influenced by the surgeon’s preference [22, 23]. In fact, even if the RP approach is burdened by the smaller working space and harder instruments handling, especially in conventional laparoscopy, many surgeons are more comfortable with such approach rather than TP in treating posterior tumors [22]. Conversely, some others underline the overcoming of these limitations with robotic technology, which augmented the surgeon’s abilities to work in a small confined space [24, 25]. Moreover, even if the RP approach requires a strong knowledge of untraditional anatomic landmarks, it offers several advantages thanks to the direct access to the renal pedicle and avoidance of the bowel mobilization.

Another paramount issue is represented by the localization of renal tumor. Indeed, tumors localized in the posterior face or in the lateral margin or lower pole of the kidney can be easily approached with a RP access. Conversely, when a TP access is preferred, a prolonged and laborious, extensive isolation of kidney from adherent perinephric fat could be necessary. Tumors of the inferior pole can be easily managed with either TP or RP PN in most of the cases. Contrarily, some cases of renal tumors located on the anterior face of the kidney can be extremely challenging with a RP access, up to become extremely risky or almost impossible to manage if these lesions are close to the renal hilum.

In our study a propensity score matching was performed to select those cases with comparable patients’ and tumor’s characteristics. The number of patients treated with RP mi-PN for anterior tumors were low (37 cases, 9%), while no case of peri-hilar tumor treated with RP mi-PN was reported. This introduces a selection bias and, again, it is mainly related to the surgeon’s belief of an easier control over tumor resection and anatomical landmarks using a TP access. Despite the conclusions of this study cannot be generalized to all the types of renal tumors, to date, no randomized trials comparing TP and RP mi-PN are available. The current evidence come from both prospective and retrospective observational studies, single and multi-institutional matched pair cohorts and few review and meta-analyses [10–13, 15, 24].

Considered together, the results of the different studies are inconclusive, being affected by confounding factors and biases in the comparison of the cohorts of patients. For example, some studies considered different sample sizes, different preoperative patient- and tumor-related features or single centers experiences [26]. Moreover, in order to avoid potential limitations, other authors focused their attention only on specific surgical approaches, like laparoscopy or robotic surgery or on specific tumor features, like posterior or complex cases [27, 28]. In a recently published systematic review and meta-analysis comparing the RP and TP approaches for posteriorly located tumors treated with RAPN, Mclean et al. found no differences in terms of perioperative outcomes except for a lower operative time in RP PN [29]. They concluded that the most suitable approach for index patients’ undergoing RAPN depends on surgeon expertise and familiarity with technique, considering the patient characteristics using a risk-stratified model.

Similar results were found by Xia et al. [30]. In their meta-analysis, they compared the perioperative outcomes of TP vs RP RAPN and showed no significant difference in terms of complication rate, conversion rate, WIT, EBL, and PSM rate. Only OT resulted marginally shorter in RP vs TP RAPN (*p* = 0.05, WMD: 28.03; 95% CI 0.41–55.65). Other two meta-analyses comparing TP and RP LPN, including 7 and 8 studies respectively, showed that RP LPN had a shorter OT and hospital stay compared with TP RAPN [10, 11]. However, many of these studies concluded that RCTs and high-quality observational cohort studies with large sample size are needed to confirm their findings.

In this study, a large heterogeneous multi-institutional population was analyzed to be as much as possible representative of the real scenario and to reduce the biases related to single centers’ or single surgeons’ settings. A main limitation of this study was the absence of the assessment of each surgeon experience. Notwithstanding with these limitations, our results suggest that center experience could be identified as a main driver of the choice of the surgical access in mi-PN. In fact, RP access was more frequent in centers performing a higher number of PN/year and with a higher PN/RN ratio per year compared to the TP group (56 vs 71 and 66.6% vs 74.0%, *p* < 0.0001). These two surrogates can be representative of the experience of the surgeons within a center.

Concerning intraoperative variables, no differences were found in terms of EBL or ischemia time, confirming results also reported by other Institutions [12, 30]. Contrarily from other available studies [16, 24], we evaluated other surgical variables potentially influenced by the surgical approach, such as the renal pedicle management and the resection technique. No significant differences between TP and RP PN emerged in terms of clampless procedures (47.9% vs 51.6%) and simple-enucleation rate (41.9% vs 40.9%).

Compared to most of the literature studies [13], we found a shorter median operative time in TP group (115 vs 150 min, *p* < 0.0001). This result could be explained considering that the RP group is composed also by robotic procedures where the operative time was longer compared to laparoscopic RP PN. In fact, in the robotic approach, the creation of the virtual retroperitoneal space is performed laparoscopically to create a working space for robotic trocars: this could determine an increase of the overall operative time.

RP approach was associated to a slightly but statistically significant lower overall and surgical intraoperative complication rates (5% vs 2.1% and 4.3% vs 1.7%, *p* = 0.03 and *p* = 0.04, respectively). These evidences can be explained considering the advantages offered by RP approach in identifying easily the renal pedicle and avoiding direct contact with the intraperitoneal organs. In fact, stratifying the surgical intraoperative complications, higher number of vascular lesions and spleen injury were recorded in TP group (9 vs 4 and 2 vs 0, respectively).

Focusing on postoperative outcomes, our findings are consistent with most of the current studies on this topic [10, 11, 15, 24]. No differences were recorded in terms of postoperative complications, even stratifying according to Clavien-Dindo classification, as well as the PSM rate and functional outcomes. Patients managed with a RP access showed a shorter postoperative recovery, with and earlier drainage removal and discharge compared to those treated with TP PN. Similar results, explainable considering a smaller fluids drainage and a faster canalization in case of RP, were found in both pure laparoscopic and robotic case series [10, 15].

To the best of our knowledge this is the largest matched pair multi-institutional study comparing TP and RP in MI-PN setting. The strength of our results is to highlight the feasibility of both approaches, offering similar intra- and postoperative results. Indeed, this highlights that, if the surgeon is confident using either the RP or the TP access, he can modulate the choice more consciously, considering all the details of each single case. Notwithstanding its strengths, the study is not devoid of limitations. First, the design of the study was not randomized, and the access of each case was determined by surgeon choice and attitude. Even if the matching minimizes the potential biases related to the comparison of different populations, a selection bias related to the lower rate of anterior and peri-hilar renal tumors in the RP group could not be corrected. Thus, definitive conclusions on which surgical access should be preferred basing on tumor nephrometric characteristics cannot be drawn. Surgeon experience was not evaluated prospectively and for this reason it could not be added as parameter for the matching balance. On the other side, all the intraoperative characteristics, such as center volume, surgical approach and clamping, were not balanced a priori to provide a comprehensive representation of the surgical management of cases according to the baseline characteristics. Moreover, no detailed long-term functional data are available and postoperative oncological outcomes were not evaluated.

## Conclusions

The results of this study suggest that both TP and RP are feasible approaches when performing MI-PN for cT1 tumors. Notwithstanding longer operative times, RP seems to have a slighter intraoperative complication rate with earlier postoperative recovery when compared with TP. Anyway, surgeons’ preference and experience remain the tips of the balance in the decision towards the surgical access while performing a MI-PN.

## Electronic supplementary material

Below is the link to the electronic supplementary material.

Supplementary file1 (JPG 1019 kb)
