# Supplementary figures and images for: Transperitoneal vs retroperitoneal minimally invasive partial nephrectomy: comparison of perioperative outcomes and functional follow-up in a large multi-institutional cohort (The RECORD 2 Project)
Source: Surg Endosc. 2020 Aug 27;35(8):4295–304. doi: 10.1007/s00464-020-07919-4 (PMC8263535; doi:10.1007/s00464-020-07919-4)

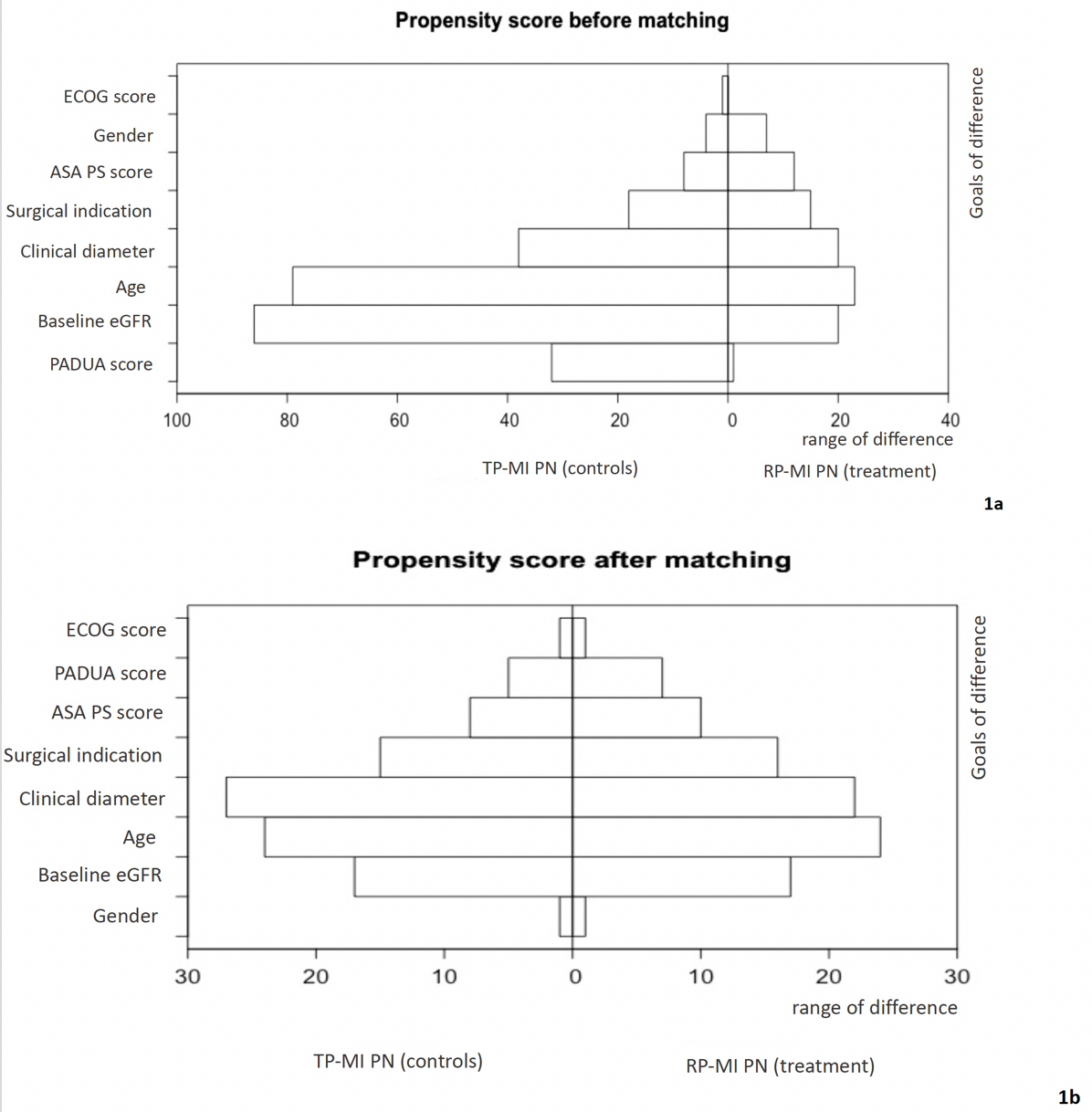

Supplement: Supplementary file 1 — Supplementary file1 (JPG 1019 kb) [file 464_2020_7919_MOESM1_ESM.jpg]
